# Integrative multi-omics database (iMOMdb) of Asian pregnant women

**DOI:** 10.1093/hmg/ddac079

**Published:** 2022-04-21

**Authors:** Hong Pan, Pei Fang Tan, Ives Y Lim, Jason Huan, Ai Ling Teh, Li Chen, Min Gong, Felicia Tin, Sartaj Ahmad Mir, Kothandaraman Narasimhan, Jerry K Y Chan, Kok Hian Tan, Michael S Kobor, Peter J Meikle, Markus R Wenk, Yap Seng Chong, Johan G Eriksson, Peter D Gluckman, Neerja Karnani

**Affiliations:** Singapore Institute for Clinical Sciences, Agency for Science, Technology and Research, Singapore; Bioinformatics Institute, Agency for Science, Technology and Research, Singapore; Singapore Institute for Clinical Sciences, Agency for Science, Technology and Research, Singapore; Bioinformatics Institute, Agency for Science, Technology and Research, Singapore; Singapore Institute for Clinical Sciences, Agency for Science, Technology and Research, Singapore; Bioinformatics Institute, Agency for Science, Technology and Research, Singapore; Singapore Institute for Clinical Sciences, Agency for Science, Technology and Research, Singapore; Singapore Institute for Clinical Sciences, Agency for Science, Technology and Research, Singapore; Bioinformatics Institute, Agency for Science, Technology and Research, Singapore; Singapore Institute for Clinical Sciences, Agency for Science, Technology and Research, Singapore; Singapore Institute for Clinical Sciences, Agency for Science, Technology and Research, Singapore; Singapore Institute for Clinical Sciences, Agency for Science, Technology and Research, Singapore; Department of Biochemistry, Yong Loo Lin School of Medicine, National University of Singapore, Singapore; Singapore Lipidomics Incubator, Life Sciences Institute, National University of Singapore, Singapore; Singapore Institute for Clinical Sciences, Agency for Science, Technology and Research, Singapore; Department of Reproductive Medicine, KK Women's and Children's Hospital, Singapore; Academic Clinical Program in Obstetrics and Gynaecology, Duke-NUS Medical School, Singapore; Academic Clinical Program in Obstetrics and Gynaecology, Duke-NUS Medical School, Singapore; Department of Maternal Fetal Medicine, KK Women's and Children's Hospital, Singapore; Centre for Molecular Medicine and Therapeutics, Department of Medical Genetics, University of British Columbia, Vancouver, BC, Canada; Department of Medical Genetics, University of British Columbia, Vancouver, BC, Canada; Metabolomics Laboratory, Baker Heart and Diabetes Institute, Melbourne, VIC, Australia; Department of Biochemistry, Yong Loo Lin School of Medicine, National University of Singapore, Singapore; Singapore Lipidomics Incubator, Life Sciences Institute, National University of Singapore, Singapore; Singapore Institute for Clinical Sciences, Agency for Science, Technology and Research, Singapore; Department of Obstetrics and Gynecology and Human Potential Translational Research Program, Yong Loo Lin School of Medicine, National University of Singapore, Singapore; Singapore Institute for Clinical Sciences, Agency for Science, Technology and Research, Singapore; Department of Obstetrics and Gynecology and Human Potential Translational Research Program, Yong Loo Lin School of Medicine, National University of Singapore, Singapore; Folkhälsan Research Center, Helsinki, Finland; Department of General Practice and Primary Health Care, University of Helsinki, Helsinki, Finland; Singapore Institute for Clinical Sciences, Agency for Science, Technology and Research, Singapore; Liggins Institute, University of Auckland, Auckland, New Zealand; Singapore Institute for Clinical Sciences, Agency for Science, Technology and Research, Singapore; Bioinformatics Institute, Agency for Science, Technology and Research, Singapore; Department of Biochemistry, Yong Loo Lin School of Medicine, National University of Singapore, Singapore

## Abstract

Asians are underrepresented across many omics databases, thereby limiting the potential of precision medicine in nearly 60% of the global population. As such, there is a pressing need for multi-omics derived quantitative trait loci (QTLs) to fill the knowledge gap of complex traits in populations of Asian ancestry. Here, we provide the first blood-based multi-omics analysis of Asian pregnant women, constituting high-resolution genotyping (*N* = 1079), DNA methylation (*N* = 915) and transcriptome profiling (*N* = 238). Integrative omics analysis identified 219 154 CpGs associated with *cis-*DNA methylation QTLs (meQTLs) and 3703 RNAs associated with *cis-*RNA expression QTLs (eQTLs). Ethnicity was the largest contributor of inter-individual variation across all omics datasets, with 2561 genes identified as hotspots of this variation; 395 of these hotspot genes also contained both ethnicity-specific eQTLs and meQTLs. Gene set enrichment analysis of these ethnicity QTL hotspots showed pathways involved in lipid metabolism, adaptive immune system and carbohydrate metabolism. Pathway validation by profiling the lipidome (~480 lipids) of antenatal plasma (*N* = 752) and placenta (*N* = 1042) in the same cohort showed significant lipid differences among Chinese, Malay and Indian women, validating ethnicity-QTL gene effects across different tissue types. To develop deeper insights into the complex traits and benefit future precision medicine research in Asian pregnant women, we developed iMOMdb, an open-access database.

## Introduction

Genome-wide association studies (GWAS) have helped identify associations between thousands of genetic variants with various diseases and traits (1). The molecular aetiologies of these phenotypes are further enhanced with molecular quantitative trait loci (QTL), linking molecular traits with phenotypes sharing genetic associations. In particular, genetic associations with gene expression and DNA methylation provide useful insight in understanding the linkage of susceptibility variants and their related genes and cell-specific regulatory elements (2). Spearheaded by international consortia such as ENCODE (3), NIH Epigenome RoadMap (4) and Genotype-Tissue Expression (GTEx) (5), the integration of genetic risk alleles to gene expression and DNA methylation profiles provides baseline references for DNA methylation and expression profiles across different tissues. Clinical insights derived from these stellar advancements in scientific knowledge are unfortunately limited by a Eurocentric bias (6) which may exacerbate the prediction of health disparities for individuals not of European descent (7). Furthermore, integrative analyses of two or more omics platforms from the same set of samples and tissue type can augment understanding of the interplay of molecular interactions far beyond the potential of single biomolecule databases (8). For example, integration of SNP, DNA methylation and mRNA expression in peripheral blood mononuclear cells provided novel insights into DNA methylation-mediated regulation effects (9). Assimilation of SNPs, RNA, DNA methylation and histone variation between distinct primary immune cell lineages yielded specific regulatory circuitry, allowing for high potential targets for experimental validation and mechanistic insight (10). Knowledge harvested from such integrative analyses can help improve precision medicine practices (11), refine pharmaceutical efficacies (12) and enhance disease prediction models (13).

Here, we provide iMOMdb, a multi-omics database of pregnant women with three predominant ethnicities of Asian ancestry, i.e. Chinese, Malay and Indian. This database provides genome-wide profiles of integrative results of meQTLs and eQTLs, as well as a suite of biomarkers differentiating these ethnic groups, including SNPs, CpGs and RNA transcripts.

## Results

### Generation of multi-omics data and its benchmarking with established resources

**Figure 1:**
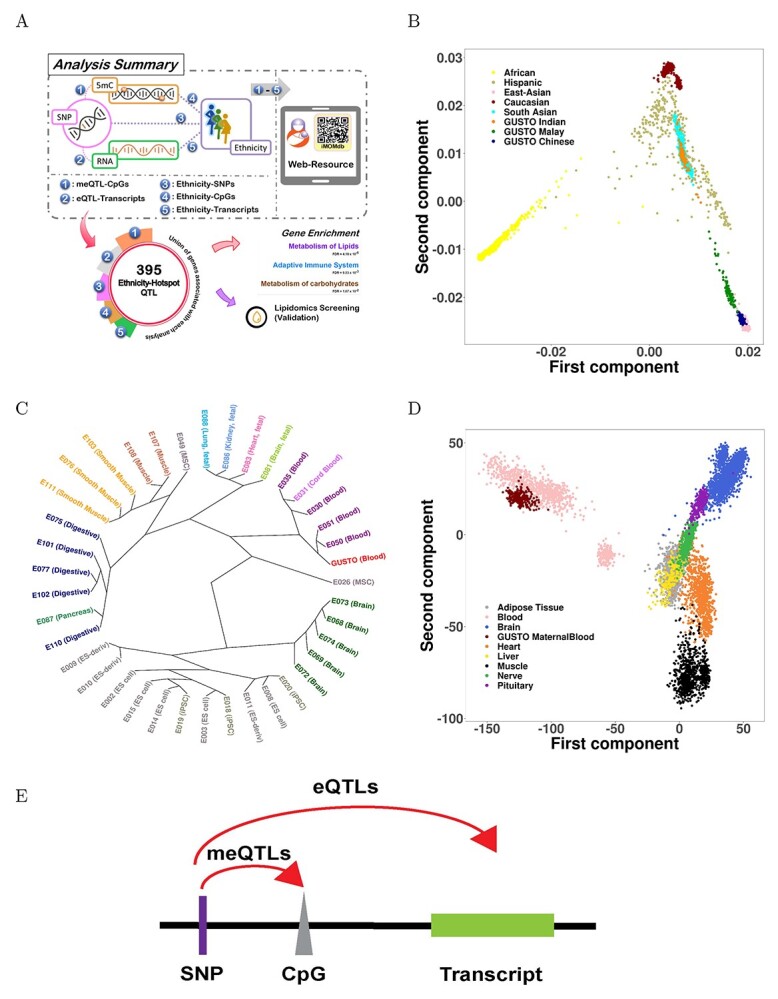
Study overview. (**A**) Graphical overview of integrative omics analysis and the iMOMdb database developed in this study. QR code included provides easy access to the database. (**B**) Benchmarking of GUSTO genotypes against the 1000 Genomes study. Scatterplot represents the 1st and 2nd principal components generated from 221 278 genetic variants merged from 1079 GUSTO maternal genotypes and 1000 Genomes (http://www.internationalgenome.org/). GUSTO Chinese and GUSTO Indians overlapped with East Asians and South Asians, respectively. GUSTO Malays formed a distinct cluster between the Chinese and Indian ethnicities but were relatively closer to East Asians. (**C**) Benchmarking of GUSTO transcriptomics data against GTEx data. Scatterplot represents the 1st and 2nd principal components generated from the merged transcriptomics data from 238 GUSTO maternal blood samples and the GTEx data available from 7567 samples covering 8 different tissue types. PCA plot shows a strong convergence between transcription profiles of GUSTO maternal blood with GTEx whole blood samples. (**D**) Benchmarking of DNA methylation data generated from GUSTO blood samples against the Epigenome Roadmap project data. The dendrogram plot shows the relationship between DNA methylation levels of CpGs profiled using EPIC arrays from 915 maternal buffy coat samples and different adult-tissue samples profiled under the Epigenome Roadmap using the RRBS method. DNA methylation profiled from GUSTO maternal blood samples clustered closely with the RRBS profiles from the blood samples available within the Roadmap dataset. (**E**) Schematic explaining *cis-*DNA methylation QTL and expression QTL (meQTL and eQTL) identified in this study.

In this study, we generated the multi-omics data from maternal antenatal blood samples collected at middle gestation in the GUSTO cohort (Fig. 1A, Supplementary Material, Fig. S1). The studied datasets included subject genotypes, DNA methylation and gene expression profiles. Genotypes of 1079 mothers were profiled using the Illumina Omniexpress + exome arrays. Quality control (QC) analysis yielded 629 493 SNPs from the arrays, and an additional 6 978 879 SNPs were imputed. DNA methylation profiles were generated (*N* = 915) using the Illumina Infinium MethylationEPICMethylationEPIC arrays, and 422 788 CpGs passed the QC (Supplementary Material, Fig. S2A). Likewise, whole genome transcriptomics was performed on the maternal whole blood samples from 238 subjects and 15 937 RNA transcripts passed QC (Supplementary Material, Fig. S2B). The ethnic breakdown of various omics platforms is provided in Table 1**.**

**Table 1:** Summary of samples and omics analysis used in this study

	**Omics**	**Tissue type**	**Method**	**Before QC**	**After QC**	**Number of subjects (Chinese/Malay/Indian)**
**Pregnancy (26-28th week)**	Genomics	Buffy coat	Illumina OmniExpress + exome array	933 886 SNPs	629 493 SNPs	1079 (615/268/196)
			Imputed SNPs	~85 M imputed SNPs	6 978 879 SNPs	
	Epigenomics	Buffy coat	Infinium MethylationEPIC Kit (EPIC 850)	866 091 CpGs	422 788 CpGs	915 (531/213/171)
	Transcriptomics	Whole blood	Whole transcriptomic, Illumina HiSeq 4000, 100 bp paired end platform	57 905 Transcripts	15 937 Transcripts	238 (146/48/44)
	Lipidomics	Fasting plasma	Liquid chromatography – Mass spectrometry (LC–MS/MS)	694 Lipid species	480 Lipid species	752 (400/198/154)
**Delivery**	Lipidomics	Placenta (maternal facing)	Liquid chromatography – Mass spectrometry (LC–MS/MS)	634 Lipid species	483 Lipid species	1042 (641/204/197)

We benchmarked our omics results against various major public data repositories. Genetic variants common between GUSTO and 1000 Genomes datasets showed genotypes of GUSTO Malays to lie between GUSTO Chinese and GUSTO Indians, though they were more closely related to the East Asians than South Asians (Fig. 1B, Supplementary Material, Fig. S3A). This result is also in alignment with a recent population study on genetic profiles in Singapore (14). Benchmarking of DNA methylation data with Epigenome Roadmap Project samples analyzed using reduced representation bisulfite sequencing (RRBS) (4) identified tissue-specific clustering, with the GUSTO maternal buffy coat samples clearly clustered within the rest of the blood samples available under the Epigenome Roadmap data (Fig. 1C). Lastly, comparison of GUSTO RNAseq transcriptomics data against the Genotype Tissue Expression Project (GTEx v8) (15) showed a strong overlap with the other whole blood samples from the GTEx dataset (Fig. 1D, Supplementary Material, Fig. S3B). Figure 1E explains *cis*-DNA methylation QTL and expression QTL (meQTL and eQTL) identified in the subsequent analysis. Also, Supplementary Material, Table S1 provides a dictionary of molecular terms used in the subsequent results.

### *Cis*-meQTL characteristics

**Figure 2:**
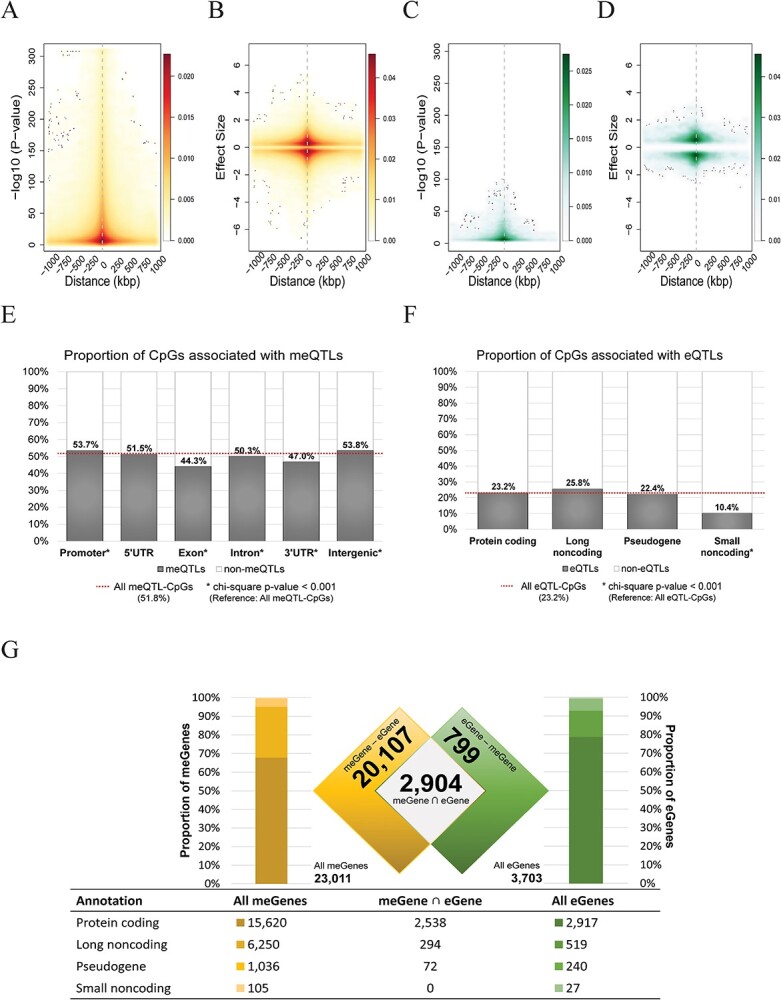
meQTL and eQTL characteristics**.** (**A**–**D**) Cloud scatter plot showing the relationship between association tests (*P*-value and normalized effect size) and *cis-*distances in base pairs. Closer the SNP is to the transcript/CpG, better is the associations in *P*-values and absolute normalized effect sizes. For 422 788 CpGs that passed QC, 219 154 CpGs passed FDR *P*-value < 0.05 in *cis*-meQTL mapping, and for the 15 937 transcripts that passed QC, 3703 transcripts passed FDR *P*-value < 0.05 in *cis*-QTL mapping. (**A**) Density plot of –log10 (*P*-value) of SNP-CpG association and the distance between them in meQTL mapping. (**B**) Density plot of absolute values of effect sizes and the SNP to CpG distance in meQTL mapping. (**C**) Density plot of –log10 (*P*-value) of SNP-Transcript association and the distance between them in eQTL mapping. (**D**) Density plot of absolute values of effect sizes and the SNP to transcript distance in eQTL mapping. (**E**) CpGs were classified based on where they lie with respect to various gene features and the proportion of CpGs which associate with meQTLs per gene feature is represented as barplots. The mean proportion of all CpGs found to associate with meQTLs is represented by a red dotted line, whereas an asterisk suffix indicates gene feature labels that were observed to be significantly different in the chi-square test. (**F**) Transcripts were classified based on their general annotation and the proportion of transcripts associated with eQTLs per annotation is represented as barplots. The mean proportion of all transcripts found to associate with eQTLs is represented by a red dotted line, whereas an asterisk suffix indicates gene feature labels that were observed to be significantly different in the chi-square test. (**G**) meGenes, genes which contain an associated meQTL, are parsed into different expression annotations (shades of yellow) alongside eGenes—genes which contain an associated eQTL (shades of green). The Venn diagram describes the relationship between the genes found distinct and common between meGenes and eGenes. The table below the barplots and Venn diagram describes the numerical breakdown of expression annotations of meGenes, the intersection between meGenes and eGenes, and eGenes.

From the 915 GUSTO subjects with both genotype and DNA methylation data, 219 154 of 422 788 CpGs (51.8%) showed significant association (adjusted *P*-value < 0.05), with a *cis-*SNP within 1 million base pairs of the same chromosome (Table 2). In general, associations of the CpG to SNP were more significant and stronger the closer the two were (Fig. 2A and B). Altogether, 75.3% of all 6 891 829 analyzed SNPs were meQTLs. Our results also revealed 22.7% of SNPs to be located within the same gene region of their related CpGs. Of the remaining meQTLs, 24.6% were intergenic and 52.7% were in a different gene region than the related CpGs. CpGs associated with meQTLs appear to be enriched in promoter and intergenic regions and depleted in genic regions such as exons and introns (Fig. 2E); 23 011 genes contained at least one CpG associated with at least one meQTL, which we termed as meGenes.

**Table 2:** Statistical summary of eQTL and meQTL analysis

	**meQTLs**	**eQTLs**
Data		
no. of subjects	915	233
no. of CpGs	422 788	15 937
no. of SNPs	6 891 829	6 790 080
Covariates	estimated cell types, ethnicity, maternal age and batches	estimated surrogate variable and ethnicity
Criteria	FDR *P*-value < 0.05, corresponding to *P*-value (median [max, min]) = 1.3 × 10^-9^ [9.2 × 10^-3^, 2.2 × 10^-308^]	FDR *P*-value < 0.05, corresponding to *P*-value (median [max, min]) = 9.5 × 10^-9^ [1.8 × 10^-3^, 6.7 × 10^-103^]
QTLs	5 190 971 SNPs	337 894 SNPs
Related genomic feature	219 154 CpGs associated with at least one meQTL; 23 011 meGenes	3703 transcripts associated with at least one eQTL; 3703 eGenes

### *Cis*-eQTL characteristics

There were 233 GUSTO mothers with both genotyping and RNA sequencing data available. Of the 15 937 transcripts that passed the QC, 3703 (23.2%) were significantly associated with a *cis*-SNP within 1 million base pairs of the same chromosome (Table 2). Most of these transcripts were protein coding (78.8%), while the rest included long non-coding (14.0%), pseudogenes (6.5%) and small non-coding (0.7%) RNAs**.** RNAs associated with eQTLs appear depleted in small non-coding regions (Fig. 2F). Of the 6 790 080 SNPs analyzed with respect to *cis*-eQTLs, only 5.0% were eQTLs; 20.7% of eQTLs were in the same gene region of the related transcripts, 18.7% were intergenic and 60.6% were in different gene regions. A similar analysis was performed using GTEx v7 whole blood eQTL data sheet (Whole_Blood.v7.signif_variant_gene_pairs.txt.gz from https://gtexportal.org/home/datasets) and found that the rates of GTEx eQTL in the same gene, intergenic region and different gene region were 17, 17 and 66%. Similar to the relationship of CpGs with their meQTLs, transcript and eQTLs were generally more significant and stronger the closer the two were (Fig. 2C and D); 3703 transcripts contained at least one eQTL, which we termed as eGenes. A large proportion of eGenes (2904, 78.4%, Supplementary Material, Fig. S2G) were also meGenes.

### Ethnic variation across multi-omics platforms

**Figure 3:**
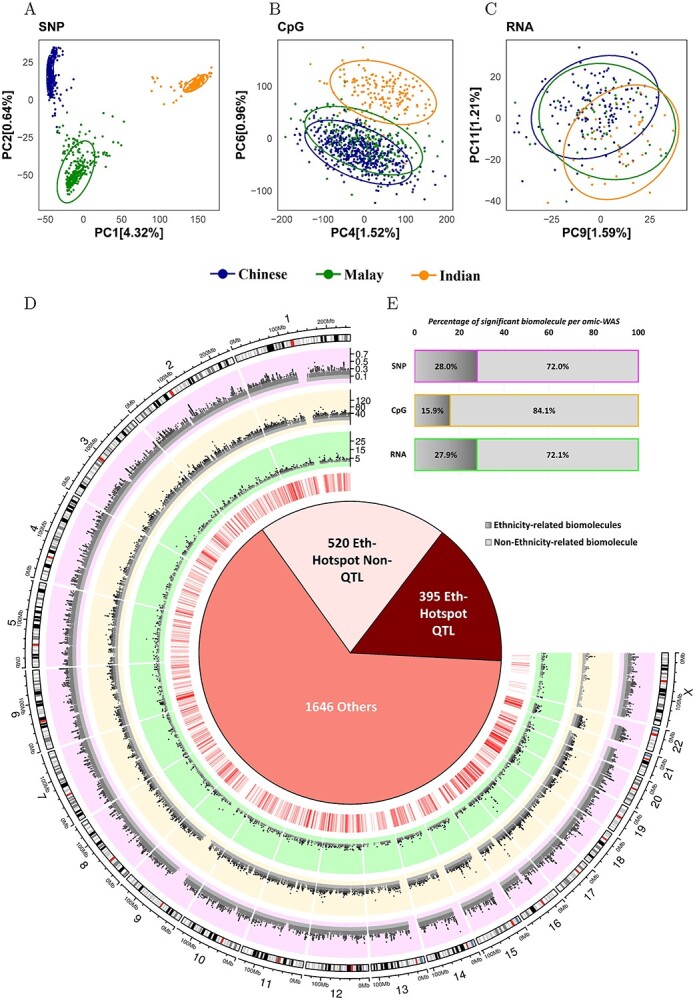
Ethnic variation across different omics datasets. (**A**) Scatter plot of 1st and 2nd principal components of 111 813 SNPs after LD pruning using imputed SNPs from 1079 subjects. (**B**) Scatter plot of 4th and 5th principal components of 422 788 CpGs from 915 subjects. (**C**) Scatter plot of 9th and 11th principal components of 15 937 transcripts from 238 subjects. Blue, green and dark orange dots indicate Chinese, Malay and Indian ethnicity, respectively. (**D**) Circos plot where outer most track shows SNPs that passed genome-wide significance for ethnicity at ${F}_{\mathrm{ST}}$ > 0.05, 2nd track shows CpGs that passed epigenome-wide significance for ethnicity analysis at FDR < 0.05, 3rd track shows transcripts that passed transcriptome-wide significance for ethnicity at FDR < 0.05 and 4th track shows all 2561 ethnicity hotspot genes (as indicated by red lines). Inner most track contains a pie chart of *N* = 395 of ethnicity QTL hotspots genes and *N* = 520 of non-QTL ethnicity hotspots genes. (**E**) Bar plot inset showing percentage of SNPs, CpGs and transcripts that were ethnicity specific.

Genetic ancestry is closely associated with ethnicity and hence plays an important role in differentiating disparate health outcomes between populations. Expectedly, principal components derived from genetics data showed clear segregations by ethnicity in this study (Fig. 3A). Principal components obtained from both DNA methylation and RNA transcript were also able to distinguish between the ethnicities (Fig. 3B and C). Ethnicity also remained the largest contributor of the recorded demographic and clinical variation in DNA methylation (1.1%) and transcriptomics (1.8%), respectively; well ahead of the second highest contributor of clinical variation (maternal age, 0.5% in DNA methylation; pre-pregnancy body mass index (BMI), 0.7% in transcriptomics, respectively; Supplementary Material, Fig. S4). Since ethnicity was the strongest contributor of inter-individual variation in the multi-omics data, we decided to explore it as a dependent variable in the subsequent analyses.

In the ethnicity specific analyses, genetic data from GUSTO mothers (615 Chinese, 268 Malay, 196 Indian), 28% (about 1.9 million) of the profiled SNPs had an ${F}_{\mathrm{ST}}$value of > 0.05 (outermost track of the Circos plot in Fig. 3D and bar plot in Fig. 3E). These SNPs mapped to 30 350 unique genes (Fig. 3D, outermost track of the Circos plot, Supplementary Material, Fig. S5, left column). Additionally, almost all (99.9%) of these SNPs passed ethnicity-based GWAS sensitivity analysis (adjusted *P*-value ≤ 0.05).

In context of DNA methylation, of the 422 788 CpGs from 915 GUSTO mothers (531 Chinese, 213 Malay, 171 Indian), 67 049 CpGs (15.9%) were significantly associated (Bonferroni *P*-value < 0.05) with ethnicity (Fig. 3D, second track of the Circos plot), corresponding to 14 629 unique genes (Supplementary Material, Fig. S5, middle column). Similar analysis targeting the transcriptomics data of the 15 937 RNA transcripts from 238 GUSTO mothers (146 Chinese, 48 Malay, 44 Indian) identified 4433 transcripts (27.8%) to be significantly associated (adjusted *P*-value < 0.05) with ethnicity (third track of the Circos plot in Fig. 3D, Supplementary Material, Fig. S5, right column), corresponding to the same number of unique genes (4433). Altogether, 2561 genes shared significant associations with ethnicity across all three omics platforms, i.e. carry at least one SNP, CpG and RNA transcript significantly associated with ethnicity (innermost track of the Circos plot in Fig. 3D, Supplementary Material, Table S2). We defined these overlapping genes as the ethnicity specific hotspot genes.

### Ethnicity hotspot genes

We further divided the 2561 ethnicity hotspot genes into sub-groups based on their association with different QTLs. Ethnicity eGenes were defined as genes related to ethnicity eQTL and its RNA expression also demonstrated ethnic differences. Similarly, ethnicity meGenes were defined as genes related to ethnicity meQTL and its DNA methylation also demonstrated ethnic differences. As such, four subgroups could be differentiated based on the permutation of ethnicity eGenes and ethnicity meGenes (Fig. 3D).

The first sub-group, termed as the ethnicity QTL hotspots, comprised of 395 out of 2561 (15%) ethnicity hotspot genes that were both meGenes and eGenes. The top three biological ontologies identified from gene set enrichment analysis (GSEA) of these 395 ethnicity QTL hotspot genes included metabolism of lipids (adjusted *P*-value 4.18 × 10^−6^), adaptive immune system (adjusted *P*-value = 9.53 × 10^−3^) and metabolism of carbohydrates (adjusted *P*-value = 1.07 × 10^−2^).

The second sub-group comprised of 520 out of 2561 (20%) ethnicity hotspot genes that did not overlap with the ethnicity eGenes or meGenes and were hence termed as non-QTL ethnicity hotspot genes. GSEA analysis identified these genes to be enriched in adaptive immune system (adjusted *P*-value = 1.17 × 10^−8^), cell cycle (adjusted *P*-value = 1.24 × 10^−7^) and cytokine signaling in immune system (adjusted *P*-value = 2.30 × 10^−7^) pathways.

The third sub-group comprised of 45 ethnicity hotspot genes that were eGenes but not meGenes (2%), and the fourth subgroup consisted of 1601 hotspot genes that were only meGenes (63%). The ethnic variation in these subgroups of genes may arise due to cell-type or epigenetic regulation-specific regulatory mechanisms (Figure 3D, pie chart, third and fourth groups collectively reported under ‘others’).

### Validation of top biological pathways highlighted by ethnicity QTL hotspots using lipidomics

**Figure 4:**
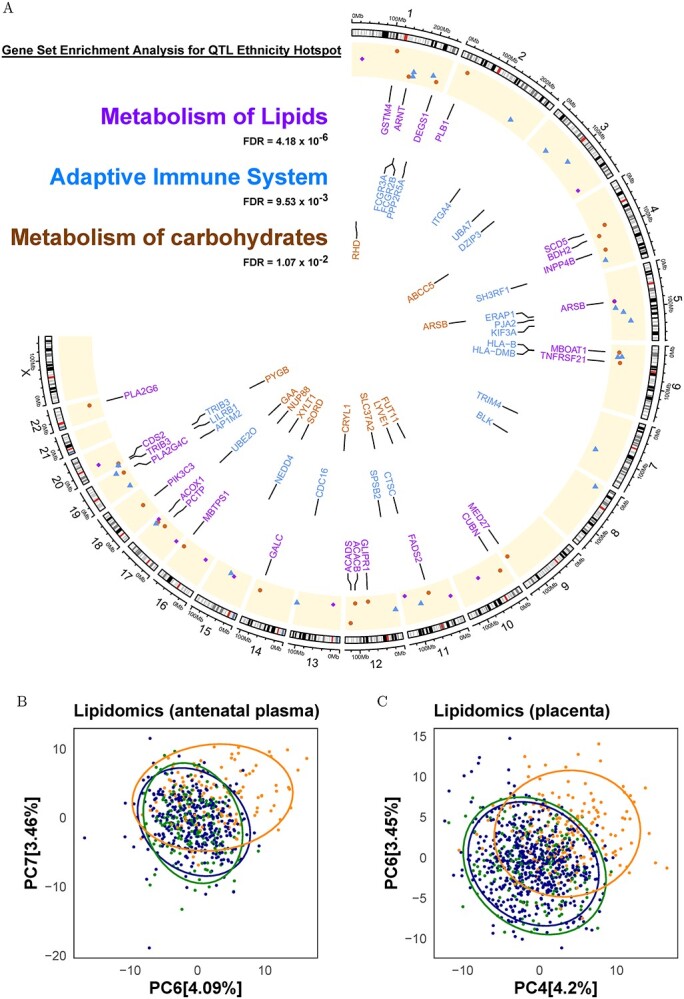
Ethnicity QTLs and enriched gene networks. (**A**) Metabolism of lipids, adaptive immune response and metabolism of carbohydrates pathways were the top 3 gene sets (FDR < 0.05) identified in the GSEA of 395 ethnicity QTL hotspot genes using REACTOME. Circos plot provides the chromosome number and name of the ethnicity QTL genes belonging to these 3 gene sets. Color coding of the gene names corresponds to the color coding of the text representing top 3 gene sets in the inset. (**B**) PCA plot of 480 lipid species measured in maternal blood plasma (*N* = 752) during mid gestation. (**C**) 483 lipid species from 1042 maternal side facing placenta samples. PCA plots were colored based on ethnicity where blue, green and dark orange dots indicate Chinese, Malay and Indian subjects, respectively.

The most significant gene set enrichment observed from ethnicity QTL hotspot genes was the metabolism of lipids (Fig. 4A). As such, we interrogated various lipid species using LC–MS/MS on fasting plasma samples of 752 GUSTO mothers to investigate whether our ethnicity QTL findings translated onto biochemical phenotypic differences; 400 of the profiled 480 (83.33%) lipid species passed the adjusted *P*-value ≤ 0.05 with respect to ethnicity, with the three most significant lipids belonging to alkenylphosphatidylethanolamine species [PE(P-18:1/20:5) at adjusted *P*-value = 2.65 × 10^−50^, PE(P-18:1/22:6) *P*-value = 5.04 × 10^−49^ and PE(P-18:0/20:5) *P*-value = 6.22 × 10^−43^] (Supplementary Material, Table S3). Principal component analysis (PCA) analysis of the plasma lipidome also reflected distinct ethnic separation in maternal blood lipid profiles (Fig. 4B).

We were further interested to investigate if the inter-ethnic differences in the lipid profiles extended beyond maternal circulation and influenced their supply to the fetus through placenta. For this, we interrogated 483 lipid species in the maternal-facing placental samples of 1042 GUSTO mothers (Fig. 4C, and Supplementary Material, Table S4). Almost 75% (360 out of 483) of the interrogated lipid species passed false discovery rate (FDR) adjusted *P*-value < 0.05, with the strongest associations including phospholipids such as the phosphatidylcholine PC(18:0_22:5) (adjusted *P*-value =1.17 × 10^−45^), phosphatidylethanolamines PE 38:5 *P*-value = 1.11 × 10^−44^ and PE(18:0_22:5) *P*-value = 7.21 × 10^−43^. Similar to antenatal plasma, PCA analysis of the placental lipidome also identified ethnic variation, with Indians showing more separation from Chinese and Malay.

### iMOMdb resource and features

Recognizing the importance and uniqueness of our multi-omics data from an Asian cohort, we developed a comprehensive online database iMOMdb of the results highlighting significant genes and variants (SNPs, CpGs, RNA transcripts, eQTLs and meQTLs), associated with ethnicity. For SNPs, ${F}_{\mathrm{ST}}$information and donut plots indicating the inter-ethnic proportions of genetic variants are provided. For DNA methylation and gene expression data, *P*-values and boxplots depicting the ethnicity differences are shown. Lastly, for meQTLs and eQTLs, associations of a CpG or transcript with a SNP along with their FDR/Bonferroni correction thresholds and whether they associate with ethnicity or not are provided.

#### User interface

The web-based interface of iMOMdb is accessible through https://imomdb.karnanilab.com/imomdb and allows users to browse, search, visualize and download data and information pertaining to multi-omics platforms and ethnic variants identified in this study).

#### Search and Charts module

**Figure 5:**
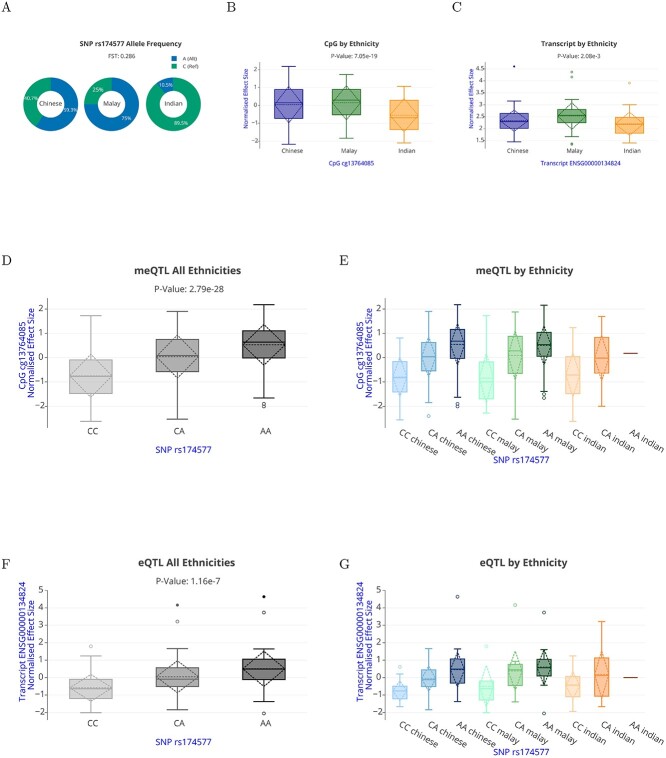
iMOMdb web-based features. iMOMdb includes eQTLs, meQTLs and ethnicity associated SNPs, CpGs and transcripts identified in this study. Data visualization available under iMOMdb is shown in **A–E** by using *FADS2* gene locus as an example. (**A**) Donut plot showing allele frequency percentage for *FADS2* SNP rs174577 within each ethnic group. (**B**) Boxplot of association between CpG cg13764085 and ethnicity (**C**) Boxplot of association between ENSG00000134824 transcript and ethnicity. (**D**) Boxplot of association between CpG cg13764085 and SNP rs174577. (**E**) Boxplot of association between CpG cg13764085 and SNP rs174577 segregated by Chinese, Malay and Indian ethnicity. (**F**) Boxplot of association between ENSG00000134824 transcript and SNP rs174577. (**G**) Boxplot of association between ENSG00000134824 transcript and SNP rs174577 segregated by Chinese, Malay and Indian ethnicity.

The user-friendly data repository of iMOMdb, provides a comprehensive overview of the multi-dimensional omics data to investigate various variants and their prevalence in Asian pregnant women. To demonstrate and highlight the features of iMOMdb, we use *FADS2*, a gene coding for fatty acid desaturase and also one of the 395 ethnicity QTL genes found in our study as an example (Fig. 5). Genetic variants in *FADS2* and other fatty acid desaturase genes mapping to 11q12-q13.1 region of chromosome 11 are known to contribute to differences in the polyunsaturated fatty acids (PUFAs) across different populations of Chinese and European ancestry (16–19). *FADS2* SNP variants are also known to have *cis-*effects on gene expression and DNA methylation levels (20).

In our dataset, the ${F}_{\mathrm{ST}}$ score of 0.286 of *FADS2* genetic variant rs174577 indicates substantial differences in the allele frequencies of rs174577 SNP across the three ethnicities. Compared with Indian, Chinese and Malay had a higher frequency of the altered allele ‘A’ than the reference allele ‘G’ (Fig. 5A). Correspondingly, EWAS and transcriptome-wide association study (TWAS) analysis also found the DNA methylation at cg13764085 and expression of *FADS2* transcript (ENSG0000134824) differed by ethnicity (Fig. 5B and C). QTL analysis showed DNA methylation at cg13764085 linked to meQTL rs174577 and differed by ethnicity (Fig. 5D and E). Likewise, gene expression of *FADS2* on ENSG0000134824 was linked to eQTL rs174577 (Fig. 5F) and variable cross the three ethnicities (Fig. 5G).

## Discussion

Our study integrated multiple omics data, including genomics, epigenomics and transcriptomics, functionally validating gene set enrichments through lipodomic analyses of two different tissue types in an Asian cohort of pregnant women. This integrative approach to study human variation is powerful and has the potential to develop deeper insights into complex traits.

We not only observed genetic diversity in the studied subject pool but also identified genetic variants that can potentially alter the DNA methylation (meQTL) and RNA expression (eQTL) status of a gene. Specifically, we identified 23 011 meGenes that carry CpGs whose methylation status is influenced by *cis*-genetic variants, and likewise 3703 eGenes whose transcription is regulated by a *cis-*genetic variant. A large proportion (78%) of eGenes identified in our study were also meGenes thereby surfacing genetic variants in Asian population that may simultaneously affect multiple molecular phenotypes and perhaps also share biological mechanisms by which the causal variant influences both expression and methylation of the same gene. For example, methylation could either respond to genetically determined variation in gene expression or it could mediate the effects of the SNP on expression (e.g. increased promoter methylation suppresses transcription factor binding).

Among the demographic and clinical phenotypes assessed in this study, ethnicity was the major contributor of inter-individual variation in all the omics datasets interrogated. There were ~2 million SNPs, 67 thousand CpGs and over 4 thousand RNA transcripts that were significantly different between the three Asian ethnic groups. These genomic loci with significant ethnic differences mapped to 2561 unique genes, which we termed as ethnicity hotspots; 395 of these ethnicity hotspot genes also possessed the whole repertoire of ethnicity-associated SNPs, CpGs, RNA transcripts, meQTLs and eQTLs—which we termed as ethnicity QTL hotspot genes. These ethnicity QTL hotspot genes were significantly enriched in biomolecular pathways, the most significant being metabolism of lipids.

Validating these purported ethnic specific differences in lipid metabolism using lipidomics platform on both antenatal plasma as well as placental tissue, our findings provide evidence that associations arising within multi-omics datasets such as our own could enhance functional outcomes that may extend beyond tissue-specific findings. Our observation on ethnic differences to be prevalent even in placenta provides new insights into the potential differences in the materno-fetal lipid crosstalk. Given the high potential of our dataset for both discovery analysis and validation of molecular phenotypes by other cohorts, we release the first version of the iMOMdb, an integrative multi-omics database, aggregating five important components, *cis*-eQTL, *cis*-meQTL and ethnicity-related SNPs, CpGs and transcripts from our cohort of 1079 pregnant women of Chinese, Malay and Indian descent. Through this database, we also provide a novel metrics to quantify the biomedical traits for Asian pregnant women.

Fixation index (${F}_{\mathrm{ST}})$ analysis of the genetics data in our study identified genomic regions with low to high frequency of genetic drift among the three Asian ethnic groups. There were 32 708 (54%) SNPs with ${F}_{\mathrm{ST}}$values in low differentiation category (0.05, 0.15), 18 529 (31%) with ${F}_{\mathrm{ST}}$ in moderately differentiated (0.15, 0.25) and 9396 (15%) with ${F}_{\mathrm{ST}}$in high differentiation $(\ge$0.25) category. Most of these ethnicity specific genetic regions were driven by allele frequency differences between Indians and non-Indians. The ethnicity-specific SNPs identified in our study also replicated all the 520 ethnicity specific regions identified in the Singapore multi-omics study in adults (21) and 25/50 (50%) SNPs (${F}_{\mathrm{ST}}$ > 0.05) included in the ancestry-informative marker panel from 1000 Genome on African, East Asian, European and South Asian populations (Supplementary Material, Table S5) (22). The lower number of ethnicity specific regions identified in the previous Singapore study could be due to the use of a relatively smaller number of subjects (*n* = 364) and SNPs (~2.5 million) used in their study.

In DNA methylation analysis, 15.9% (67049) of the CpGs showed inter-ethnic variation in the levels of methylation. This differential methylation among the three Asian sub-groups could potentially arise because of genetic ancestry or shared environmental exposures. Recent studies have shown that almost 20% of the reliably assayed variation in blood DNA methylation is heritable and that 50% of CpG sites show evidence of a significant genetic component (23). Our own previous work on variably methylated regions between neonates of Asian ancestry found 25% of these variable regions to be best explained by genotype alone and 75% by a combination of genotype and environment (24). Population comparison studies, such as those done between African and European populations, have identified 77 857 CpGs (23) that are more genetically distinct (${F}_{\mathrm{ST}}$ > 0.1). Likewise, a similar study done on 573 Latino children of diverse Latino sub-ethnicities enrolled in the Genes-Environment and Admixture in Latino Americans (GALA II) study also identified methylation differences at 916 CpGs to be associated with self-reported ethnicity and 194 CpGs by genetically determined ancestry (25). Hence, such methylation differences postulated to have originated from genetic or environmental influences unique to populations or geographical regions, also bear the potential of ethnicity prediction from DNA methylation data (26).

In addition to methylation, there is growing body of evidence indicating that ethnicity can also impact gene expression. For example, in a study on 270 individuals derived lymphoblastoma cell lines from the HapMap consortium, Stranger *et al.* (27) showed that gene expression levels are hereditable and indicated an abundance of *cis*-regulatory variation in the human genome. In an independent study of individuals of European and African ancestry, Storey *et al.*(28) estimated that ∼17% of genes are differentially expressed among populations. Our own findings from this study show expression profiles of 27.8% transcripts to vary by ethnicity among Asian women; 47 of these ethnic variants were also observed to vary in the whole blood transcriptome profile of African, American and European populations (29) (Supplementary Material, Table S6).

In the QTL analysis, meQTLs identified in GUSTO pregnant women showed moderate to high replication in independent studies. For example, there was 66% concordance with the Framingham Heart Study (30), 72% with the study from Sweden people (31) and 87.5% with the Brisbane Systems Genetics Study and the Lothian Birth Cohorts (combined *n* = 1366) (32). For eQTLs, reproducibility among different ethnic groups and tissues is known to vary (5) with tissue-specificity exerting a stronger influence compared with genetic ancestry. For example, in our study consisting of relatively healthy pregnant women, we identified 3703 eGenes, of which 82.7% (3603) overlapped with GTEx whole blood data (5) derived from a predominately male, relatively older, Caucasians and African Americans cohort (irrespective of the direction of effect size). For the remaining 640 eGenes that did not overlap, 290 genes were not available in the GTEx data, and 350 were studied but did not pass significance in GTEx whole blood analysis. A comparative summary of the outcomes of this study with existing publications is provided in Supplementary Material, Table S7.

Our study has a few limitations. We had a disproportionate number of subjects in the three ethnic groups, with Chinese being overrepresented. However, the sample size of Indian and Malay were comparable to most multi-omics studies reporting inter-ethnic variation. Also, we reported only the findings that passed a stringent statistical cut-off recommended under each omics field. Likewise, for individual omics profiles, we had a relatively smaller sample size for RNA expression analysis (*N* = 238), but this sample size was on the higher side of what is typically used in such an analysis. For example, GTEx recommends a sample size of at least 70 samples per tissue to provide sufficient statistical power for eQTL discovery (5).

The major strength of this study is the establishment of iMOMdb, an open access multi-omics resource that provides useful insights into the biological information at multiple levels that can help unravel the mechanisms underlying biological condition of interest. Here, we used ethnicity as an example of a phenotype that distinguishes molecular phenotypes and complex interactions between them, but this data can be further explored to address clinical needs, such as the roles these molecular phenotypes play in chronic disease, pregnancy outcomes and aging. Our own observations from the PCA analysis of the methylation and expression data showed age and BMI to be significantly linked with molecular variation, thereby highlighting the potential of the iMOMdb in facilitating future clinical research in multiple avenues. iMOMdb also has additional features that enables researchers to extend their candidate molecular variant analysis to other well-known data resources in the omics field, such as SNPedia, Ensemble, Geography of Genome Variant (GGV) Browser, gnomAD, UCSC and NCBI. iMOMdb can also be accessed on mobile devices by use of a QR code (Fig. 1A).

In summary, multi-omics data structures are growing quickly and are being deployed for genomics medicine. The unique compendium of datasets generated in this study and its open access provides multiple opportunities for future research in precision medicine field.

## Material and Methods

### Growing Up in Singapore Towards Healthy Outcomes cohort

This study is part of the Growing Up in Singapore Towards Healthy Outcomes (GUSTO) study, a population-based prospective cohort study (33); 1247 pregnant women between 18 and 50 years of age were recruited from the two major public hospitals in Singapore, including National University Hospital (NUH) and KK Women’s and Children’s Hospital (KKH), between June 2009 and September 2010. Participants had to be Singaporean citizens or permanent residents, of Chinese, Malay or Indian ethnicity, with parents of homogenous ethnic background, had intention to deliver in NUH or KKH, had planned to reside in Singapore 5 years post-recruitment and were willing to provide biosamples (e.g. blood and placenta) during the course of the study. Here, we define ‘ethnicity’ as the classification of these distinct genetic subgroups of Asian descent. Furthermore, in our QC checks, we confirmed the segregation of differing ethnic groups with genotype and principal component analyses. Mothers receiving chemotherapy, psychotropic drugs or who had type I diabetes mellitus were excluded (33). Interviewer administered questionnaires were used to assess maternal self-reported pre-pregnancy weight, maternal age, obstetric and medical history during enrolment. Gestational weight gains (GWG) up to 26th–28th weeks of pregnancy were calculated by subtracting self-reported pre-pregnancy weights from weights measured at 26th–28th weeks of gestation. BMI was calculated from weights divided by height squared (kg/m^2^). The participants also underwent a 2-h 75 oral glucose tolerance testing (OGTT), including fasting blood samples collected at 26th–28th weeks of gestation. Sample and subject characteristics are summarized in Table 1 and Supplementary Material, Table S8, respectively.

### Genotyping and QC

#### Illumina human OmniExpress + exome genotyping array

Genomic DNA was extracted from the buffy coat available at 26th–28th weeks of gestation from 1079 participants in the study. Ethnic distribution of the participants was 615 Chinese, 268 Indian and 196 Malay. A genome-wide scan of 933 866 tagging SNPs was conducted using Illumina Omniexpress + exome arrays (Illumina, San Diego) which was performed by the service provider, Expression Analysis Inc. Data were processed in GenomeStudio Genotyping Module™. Genotype calling was done using GenCall software (Illumina, San Diego, CA), which uses GenTrain clustering algorithm and Bayesian model calling algorithm. Genotypes with a Gen Call score less than 0.15 were not considered. Principal components analysis was used to confirm self-reported ethnicity/ancestry. Samples with call rate <95%, cryptic relatedness and sex/ethnic discrepancies were excluded. QC was performed separately for each ethnicity group using PLINK v1.9 (34). SNPs with call rates <95%, minor allele frequency (MAF) < 5% or those that failed Hardy–Weinberg Equilibrium *P*-value (*P*_HWE_) < 1.0 $\times$ 10^−6^ were excluded from the analysis (35,36), and 629 493 autosomal SNPs were available for imputation. Alleles on the positive strand were reported based on the hg19 human genome assembly.

#### SNP imputation

SNP imputation was conducted using the Phase 3 Asian panel from 1000 Genomes Project (37), for Malay we used the EAS (i.e. Chinese, Japanese, Vietnamese) with a more lenient tolerance level. Software package shapeit (38) and Impute2 software packages (38,39) and SNPs were performed under the hg19 assembly. A total of 29 million SNPs were imputed after removing SNPs with imputation quality *R*^2^ < 0.5, MAF < 0.05, genotype call rate < 95% and P_HWE_ < 1.0 $\times$ 10^−6^. Overall, 6 978 879 imputed bi-allelic variants passed the QC in at least one ethnicity (Chinese, Malay, Indian) (40) and were used in this study.

### DNA methylation and QC

DNA methylation profiling on 915 maternal buffy coat samples was performed using Infinium MethylationEPIC BeadChip. DNA methylation IDAT files were processed in R using the *minfi* package (41). Probes with fewer than three beads for either the methylated or unmethylated channel, or with detection *P*-value ≥0.01, were removed. Probes on Y chromosomes, cross-hybridizing probes (42) and probes with SNPs at the CpG site or its single-base extension were excluded. Within-sample normalization was performed using Noob pre-processing (43). The beta values were first converted to M-value to perform ComBat (44) for removing chip effects. The adjusted M-values were then converted back to beta values for analysis. Finally, we removed probes where the DNA methylation range (maximum-minimum, excluding outliers) was less than 5%. In total, 422 788 CpGs passed the QC criteria. Genome coordinates (hg19 build) and gene annotations of these CpGs were extracted from the Infinium MethylationEPIC BeadChip manifest file. Cellular proportions were estimated using a cell-type specific panel (45) and included as technical covariates in subsequent models.

### Benchmarking GUSTO cohort DNA methylation data against Epigenome Roadmap

Thirty-eight primary tissues/cells profiled using RRBS from the Epigenome Roadmap project (4) were downloaded and processed for benchmarking analysis. Briefly, reads from both strands of the Epigenome Roadmap were combined, and only those CpGs were retained that had a minimum reads coverage of 30X, were not missing more than 10/38 Epigenome Roadmap samples and had interquartile range greater than 15% across different Epigenome Roadmap tissues/cells. For GUSTO samples, the median DNA methylation value across all 915 samples was used to represent each CpG. Finally, hierarchical clustering was performed on the combined GUSTO and Epigenome Roadmap CpG dataset.

### RNA-seq transcriptomics and QC

Total RNA was extracted from the whole blood samples available at 26th–28th weeks of gestation from 238 participants in the study. Whole transcriptome libraries were constructed according to Illumina’s protocol and quantified by real-time polymerase chain reaction (real-time PCR). Sequencing was performed using the Illumina HiSeq 4000 system. Raw sequencing reads were first assessed using FastQC v0.10.1 (http://www.bioinformatics.babraham.ac.uk/projects/fastqc) and reads with low-quality scores and short sequences of less than 50 bases were removed using Trimmomatic v0.36 (46). Surviving reads were aligned against GRCh37 human genome assembly (Ensembl) using STAR (version 2.7.2c) (47) and unique reads were quantified using *–quantmode* function embedded within STAR. Sample normalization was performed using Bioconductor packages in *edgeR* (48) and *limma* (49). Briefly, read counts from 238 subjects were read into R and transcripts with an average read count of less than 5 and counts per million (CPM) of less than 1 in at least 50 samples were removed, and 15 937 transcripts remained after QC. Next, to account for library size differences, a Trimmed-Mean of M values (TMM) normalization, implemented in the *calNormFactors* function within the edgeR package, was performed. One sample was removed due to the missing ppBMI value. Normalized log CPM values were then extracted and used for further QTL analysis.

To account for possible technical covariates such as sample batch and cell-type heterogeneity, surrogate variables were identified and estimated using unsupervised SVA via SVAseq package (50) (version 3.34.0) from R adjusting for pre-pregnancy BMI, fasting and 2-h post-load glucose levels and gestational age.

Since SVA does not allow for missing data, missing pre-pregnancy BMI values were replaced with booking BMI values due to their high correlation value (Pearson correlation = 0.962). Using the num.sv function with default setting (method = ‘be’), surrogate variable was estimated and used as covariate for all subsequent analysis.

### Benchmarking GUSTO cohort transcriptomics data against GTEx

GUSTO maternal whole blood RNA expression data were compared against the whole blood data from GTEx portal (15). Gene read count data (v8) as well as data dictionary containing information about sample attribute on the GTEx website, https://gtexportal.org/, for 7567 samples consisting of 8 distinct tissue types including liver, heart, adipose tissue, nerve, brain, pituitary, muscle and blood were sub-selected to be included for further analysis. Using the *limma* and *edgeR* package in R, read counts from 238 GUSTO and 7567 GTEx samples were read and merged. Any transcripts with an average read count >5 and CPM >5 in at least 5000 samples were retained. Next, TMM normalization was performed using the calcNormFactor function to account for library sizes differences. Normalized log CPM values were then extracted and subjected to PCA analysis. Using similar approach as the genomics platform, the 1st, 2nd and 3rd principal components were used to investigate the closeness between GUSTO maternal whole blood data against GTEx whole blood data as well as other tissue types.

### Lipidomics

#### Antenatal maternal plasma

Antenatal maternal plasma samples were collected during 26th–28th weeks of gestation and prepared for lipidomics analysis as described previously (51,52). Briefly, lipid extraction was carried out from 10 μL of plasma by adding 100 μL of butanol: methanol (extraction solvent) in a ratio of 1:1 (v/v) containing 10 mM ammonium format and lipid class specific internal standards. Next, the samples were vortexed for 10 s followed by sonication for 60 min with temperature maintained at 18–22°C. The samples were centrifuged at 13 000 g for 10 min. The supernatants were collected in mass spectrometry compatible vials and stored at −80°C for LC–MS/MS. These lipid extracts were analyzed by using Agilent 6490 QQQ mass spectrometer interfaced with an Agilent 1290 series HPLC system. Lipid extracts were separated on a ZORBAX RRHD UHPLC C18 column (2.1 × 100mm 1.8 mm, Agilent Technologies, 5301 Stevens Creek Blvd Santa Clara, CA 95051 United States) with the thermostat set at 45°C. Mass spectrometry analysis was performed in ESI positive ion mode with dynamic multiple reaction monitoring (53,54). QC samples (prepared by pooling study samples) and blanks were processed and analyzed along with the study samples within each batch. QCs were used to correct signal drift introduced by sample preparation and experimental measurements across batches. After removal of 11 outlier samples by PCA, a total of 480 lipid species representing 25 lipid classes (*N* = 752) were used in this study.

#### Lipidomics of maternal side facing placenta

Maternal-facing placenta samples were collected, stored and homogenized using the previously reported lipid extraction and analysis methods (51,52,54). Briefly, lipids were extracted from 20 μL (~100 μg of protein) of placental homogenate using chloroform/methanol (2:1 (v/v), 20 volumes). Lipid analysis was performed by liquid chromatography, electrospray ionization-tandem mass spectrometry using an Agilent 6490 triple quadrupole mass spectrometer interfaced with Agilent 1290 liquid chromatography system and the lipid extracts were separated on a 2.1 × 100 mm Zorbax Eclipse Plus 1.8 μm C18 column. The relative concentration of each lipid species was calculated from the peak area of the lipid species normalized to the corresponding internal standards. Placenta QCs were used for batch correction. No extreme outliers were detected by PCA; 1042 maternal-facing placenta samples were analyzed.

### *Cis*-Qualitative trait loci mapping

#### Cis-QTL mapping

*Cis*-QTL mapping was conducted using QTLtools (55) (version 1.0) and the procedure is illustrated in Supplementary Material, Figure S6, which is similar to that performed in EyeGEx (56). In *cis-*eQTL, the mapping window was defined as 1 Mb up- and down-stream of the transcript start site and end site, and the analysis was performed on 233 GUSTO subjects for 15 937 transcripts and 6 790 080 genotyped and imputed SNPs were available post-QC. In *cis-*meQTL, the mapping window spanned ±1 Mb region centered by the CpG locus. The identification of *cis*-meQTLs was performed on 915 GUSTO subjects for the post-QC 422788 CpGs and 6 891 829 genotyped and imputed SNPs. *cis*-QTL mapping was conducted in two phases. First, the nominal *P*-value of the association between SNP to the continuous traits (gene expression and DNA methylation) was conducted by the QTLtools *cis* function adjusted by ethnicity and a surrogate variable described in the section above. Second, to account for multiple tests, an adaptive permutation mode was used with the setting—permute 1000 for each *cis*-association. A FDR was estimated under the *q*-value paradigm (28) by the hypothesis of beta distribution-extrapolated empirical *P*-values. An adjusted *P*-value <0.05 was applied to identify genes related to at least one significant *cis-*eQTL (‘eGenes’) or *cis-*meQTL (‘meGenes’). We note that although no database of meQTLs exists to benchmark our findings, most of our maternal blood *cis*-eQTLs (at about 71%) were present in GTEx whole blood analysis. The summary for QTL analyses is provided under Table 2**.**

#### Covariates for QTL analysis

To control for population effects on the discovery of QTLs, ethnicity information was used as a covariate in both meQTL and eQTL mapping. For eQTL mapping, a surrogate vector for batch effect by SVA analysis (described in above section) is also included. In order to test the robustness of *cis*-eQTL discovery, pre-pregnancy BMI, maternal age and 2-h post-load glucose level were added to the main confounders in sensitivity analysis for eQTL mapping; 96.0% eQTL replicated in the sensitivity study, of which 95.7% of the related SNPs were replicated. For meQTL mapping, covariates include maternal age, chip position, DNA extraction method, hospital for blood collection and cellular composition. In sensitivity study, pre-pregnancy BMI and 2-h post-load glucose levels were added to the main confounders and the sample size reduced to 816 for the completeness of the variates for meQTL mapping. Results showed that the *cis*-meQTL mapping was robust after modifying the covariates and sample size. Sensitivity analysis replicated 96.4% meQTLs and 96.7% of the related CpGs from the primary study (Supplementary Material, Fig. S7 and Supplementary Material, Table S9).

#### Maternal clinical phenotypes and inter-individual variation in multi-omics data

PCAs was performed on genetics, DNA methylation and transcription data independently. Using genomic data, PCA was performed with PLINK1.9 using a subset of 111 813 SNPs after linkage disequilibrium pruning of 629 K genotyped SNPs from 1079 subjects. For DNA methylation and transcriptomics data, PCs were generated using 422 788 probes with >5% variation in 915 samples and 15 937 transcripts from 238 subjects, respectively. The summation of adjusted R-square values obtained from univariate linear regression of clinical phenotypes against all principal components of each omics platform (genetics, DNA methylation and transcriptomics) was performed to obtain the proportion of variation explained per clinical phenotype. These clinical phenotypes include ethnicity, pre-pregnancy weight, height, pre-pregnancy BMI, fasting and 2-h post-load glucose levels as well as GWG (Supplementary Material, Fig. S4). Data showed that among the eight studied variables, ethnicity contributed the greatest variability among the eight covariates within the omics platforms investigated. Similar PCA analysis was also conducted on lipidomics data of antenatal plasma (*N* = 752) and placenta (*N* = 1042) at delivery (Fig. 4B and C).

### Ethnicity-based multi-omics association studies

#### Genome-wide association study with ethnicity

To investigate the inter-ethnic variation in genotypes, Weir–Cockerham estimator (57) was used to calculate the ${F}_{\mathrm{ST}}$ for Chinese, Malay and Indian subgroups. ${F}_{\mathrm{ST}}$ is a widely used measure to quantify the extent to which allele frequency is different between ethnicity/ethnic groups at each genetic variant (21,58). This was measured by using the formula below. To investigate the inter-ethnic variation in genotypes, Weir–Cockerham estimator (57) was used to calculate ${F}_{\mathrm{ST}}$ for Chinese, Malay and Indian subgroups. ${F}_{\mathrm{ST}}$ is a widely used measure to quantify the extent to which allele frequency is different between ethnicity groups at each genetic variant (21,58). This was measured by using the following formula:

$$ {F}_{\mathrm{ST}}=\frac{\left(k-1\right).{\sigma}^2}{k.\overline{p}.\left(1-\overline{p}\right)} $$

Here, ${\sigma}^2$ indicates the variance frequency of a particular SNP, $\overline{p}$ indicates the average frequency of the same allele in the cohort and *k* indicates the total number of sub-populations. In general, ${F}_{\mathrm{ST}}$values were calculated to reflect on the joint effects of drift, migration, mutation and selection based on the distribution of genetic variation among populations (58), where a bigger ${F}_{\mathrm{ST}}$value indicates a greater difference in the allele frequencies across population. The ${F}_{\mathrm{ST}}$ calculation was conducted by vcftools (option –weir-fst-pop) (59). The ${F}_{\mathrm{ST}}$ values between two populations were estimated by the average value from single SNP, which was defined as the ‘average of ratios’ in (60). An SNP was considered significant if ${F}_{\mathrm{ST}}$ > 5% (21), there were 1.9 million ethnicity-related SNPs, corresponding to 30 350 gene regions (Supplementary Material, Fig. S5).

#### DNA methylation associations study with ethnicity

The relationship between DNA methylation and ethnicity was analyzed via multivariate regression analysis adjusted for maternal age, chip position, DNA extraction method, hospital for blood collection and cellular composition. Pre-pregnancy BMI and 2-h post-load glucose levels were added on top of the main covariates in sensitivity analyses. A probe was considered significant if the adjusted *P*-value was below the Bonferroni threshold in both multivariate and univariate analyses (*P*-value < 1.18 × 10^−7^) (Supplementary Material, Fig. S5).

#### TWAS with ethnicity

Differential analysis for ethnicity was performed using an empirical Bayes linear fit model approach (after Voom to account for mean–variance relationship) adjusting for surrogate variables. For this analysis, contrast matrix between each pair of ethnicity group was set. The overall *P*-values and the resulting log fold-change values for each ethnicity pairwise comparison were then obtained by using the *topTableF* function embedded within the Limma package. Similar to the EWAS study for ethnicity, BMI, OGTT 2 h and maternal age were included in addition to the main covariates for sensitivity analysis. Finally, nominal *P*-values were adjusted for multiple testing using Benjamini–Hochberg (BH) correction. A linear mRNA transcript was considered significant if the adjusted *P*-value was less than 0.05 (Supplementary Material, Fig. S5).

#### Association of antenatal plasma and placental lipids with ethnicity

We tested the association of antenatal plasma and placental lipids with ethnicity by performing a one-way ANOVA analysis on the log10 transformed lipidomics data. Altogether, 480 and 483 lipid species were tested for antenatal plasma and placenta lipids, respectively. We considered a lipid species to be significantly associated with ethnicity if the BH-corrected FDR adjusted *P*-value <0.05 for both antenatal plasma and placenta lipid species (Supplementary Material, Tables S3 and S4).

### Gene annotation

Gene annotation is based on GRCh37 human genome assembly Homo_sapiens.GRCh37.87.gtf build. The information for gene identification, start site end site and biotypes was used in eQTL mapping and related genomic annotation. In order to merge ethnicity-related SNPs, CpGs and transcripts, a gene region was defined as 5000 base pair up and downstream of the gene start and end site, respectively. Using ‘bedtools intersect’, the gene regions where the ethnicity-related SNPs, CpGs, transcripts co-located were defined as ethnicity hotspots. If an ethnicity hotspot overlapped with both eGenes and meGenes, we defined the gene region as ethnicity QTL hotspot gene (Supplementary Material, Fig. S6).

### Gene set enrichment analysis

GSEA was performed using Molecular signatures Database version 7.0 (MsigDB, CP:Reactome set) in GSEA (61). The *P*-value is calculated using hypergeometric distribution based on the number of overlapped genes associated with the number of the genes in the gene set, taking into consideration all observed genes. The FDR adjusted *P*-value was applied to adjust for multiple testing. A pathway was considered significant if the total gene counts within a gene set did not exceed 1000 and passed an FDR adjusted *P*-value < 0.05 (Supplementary Material, Fig. S8).

## Supplementary Material

Supplementary_Figure_HGM_ddac079

Supplementary_Table_S1_ddac079

Supplementary_Table_S2_ddac079

Supplementary_Table_S3_ddac079

Supplementary_Table_S4_ddac079

Supplementary_Table_S5_ddac079

Supplementary_Table_S6_ddac079

Supplementary_Table_S7_ddac079

Supplementary_Table_S8_ddac079

Supplementary_Table_S9_ddac079

## Data Availability

All raw and processed RNA sequencing data as well as the DNA methylation data generated in this study have been submitted to the NCBI Gene Expression Omnibus (GEO; https://www.ncbi.nlm.nih.gov/geo/) under the accession numbers GSE182409 (Corresponding Reviewer token number: qjolmmeudnofnsv) and GSE158063, respectively. iMOMdb provides open access to eQTLs, meQTLs and ethnicity specific SNPs, CpGs and transcripts. Placental and antenatal blood lipids data are provided as supplementary data files. Due to ethical concerns, clinical data cannot be made openly available. However, the GUSTO study team can provide such data upon request, subject to appropriate approvals after a formal application to its executive committee.
