# Supplementary material for: Integrative multi-omics database (iMOMdb) of Asian pregnant women
Source: Hum Mol Genet. 2022 Apr 21;31(18):3051–67. doi: 10.1093/hmg/ddac079 (PMC9476622; doi:10.1093/hmg/ddac079)
Supplement: Supplementary_Figure_HGM_ddac079 [file supplementary_figure_hgm_ddac079.pdf]

iMOMdb

Supplementary Figures

March 15, 2022

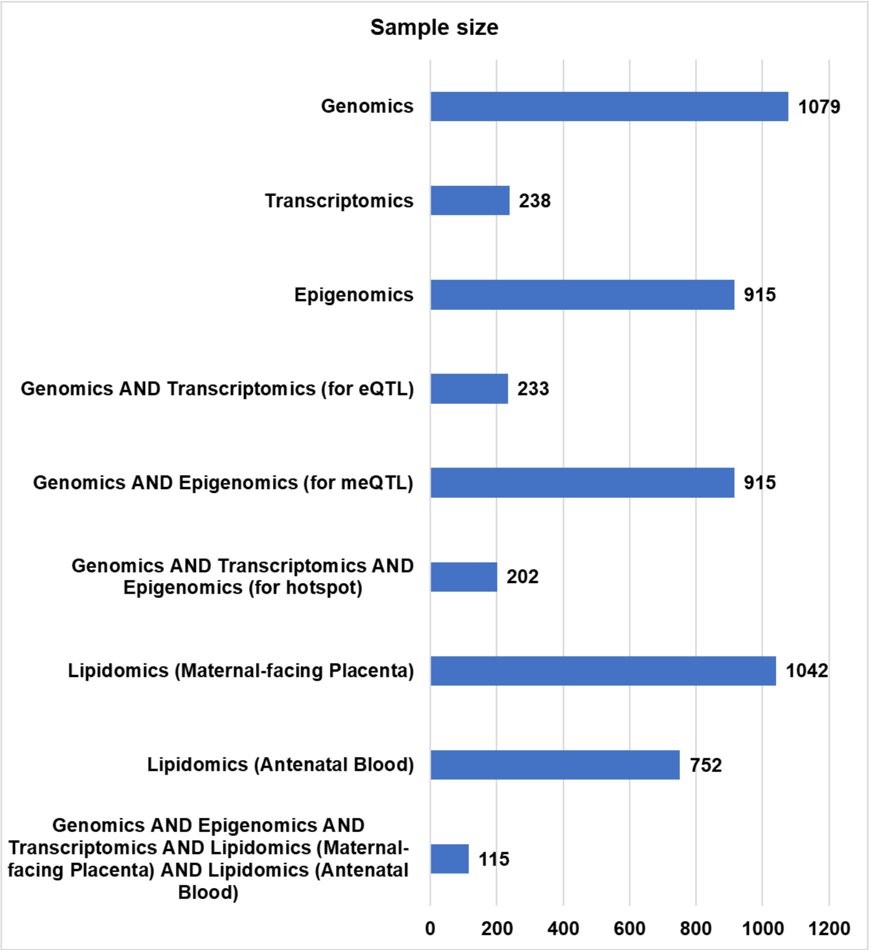

Supplementary Figure 1: The number of participants in omics data. We used "AND" to indicate the overlapping samples cross different datasets.

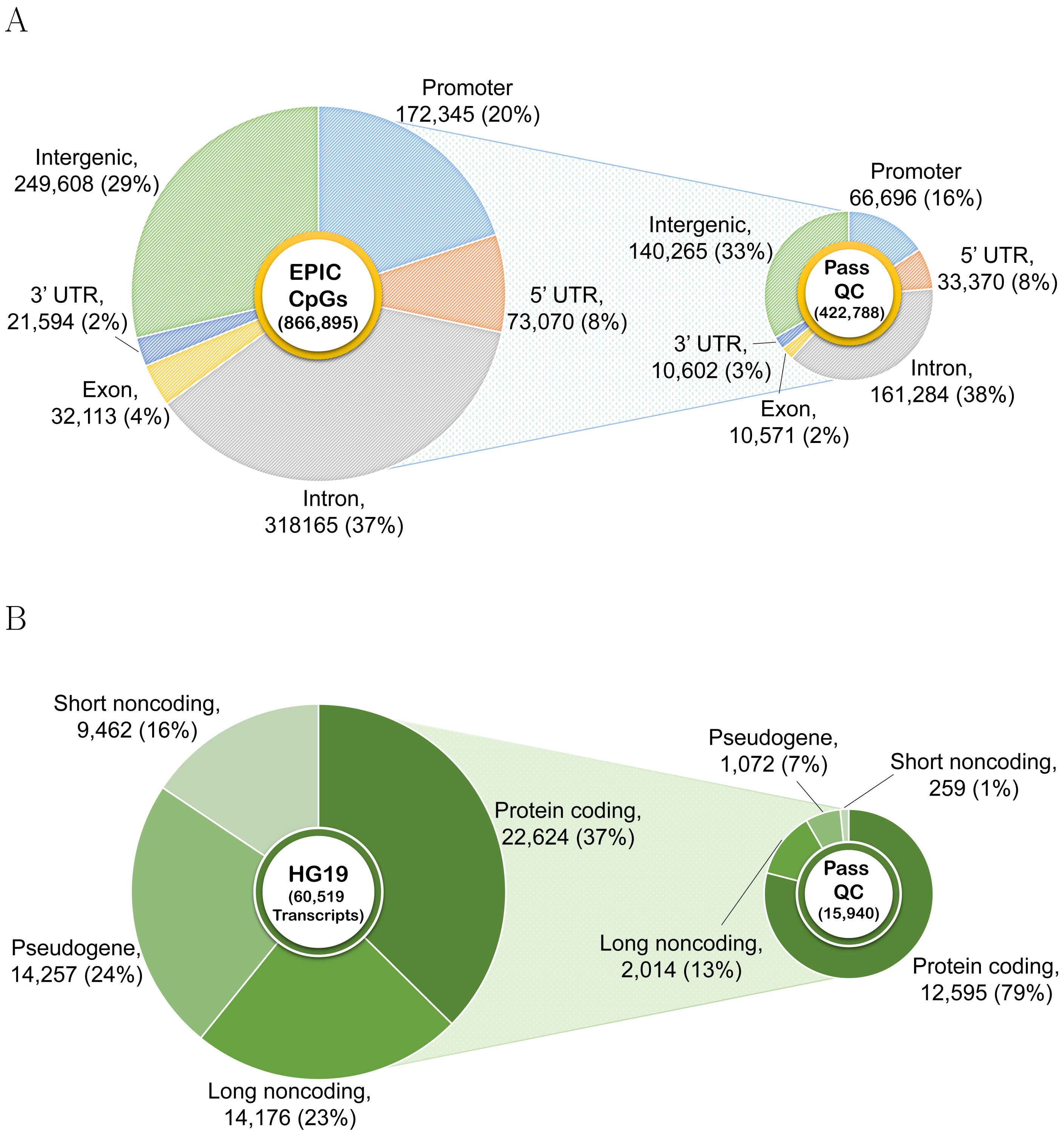

Supplementary Figure 2: The genomic annotation for **(A)** DNA methylation from EPIC platform and **(B)** transcripts from RNA-seq before and after quality control.

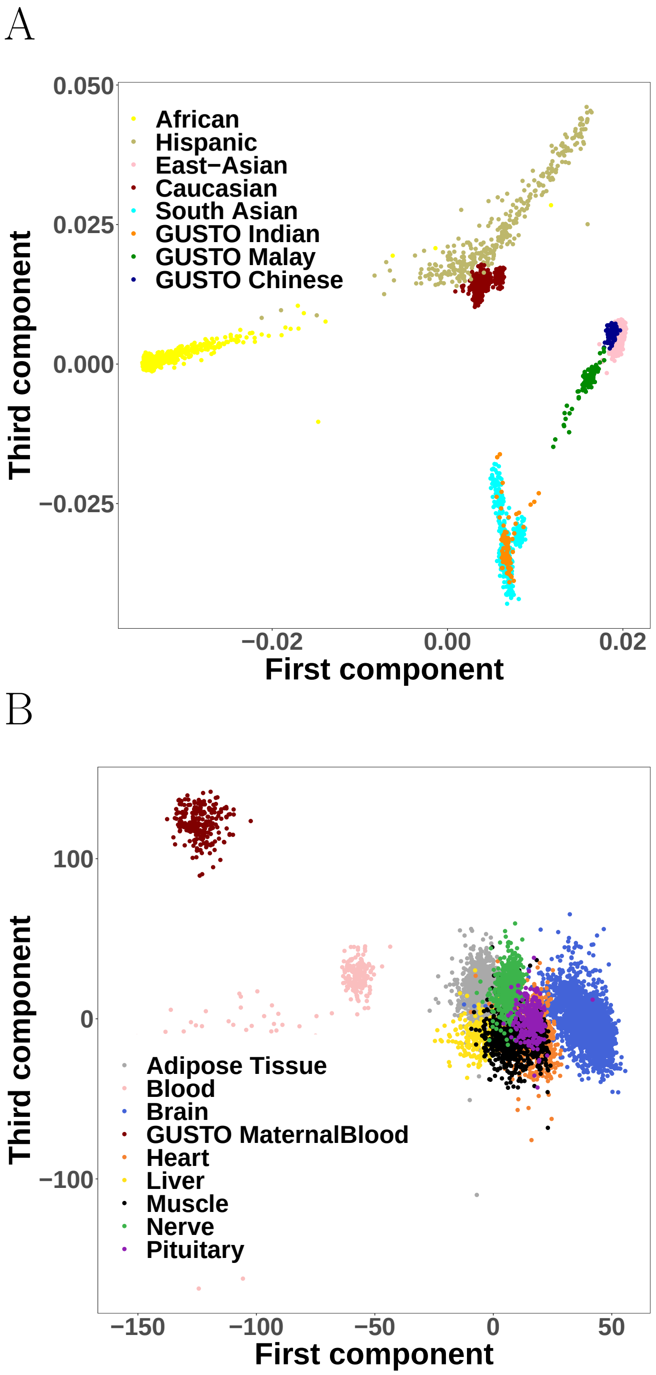

Supplementary Figure 3: **(A)**. Genetic benchmarking of GUSTO samples against 1000 Genomes. This scatterplot represents the 1st and 3rd principal component generated from the merged 221,278 genetic variants from 1079 GUSTO maternal genotypes and 1000 Genomes. Data suggests GUSTO Chinese are related to East Asian, while GUSTO Indians are more related to South Asian. GUSTO Malays are in between but more closely related to East Asians. **(B)**Transcriptomic benchmarking of GUSTO samples against GTEx. This scatterplot represents the 1st and 3rd principal components generated from the merged transcriptomics data from 238 GUSTO maternal bloods and 8 tissue types in GTEx based on 7567 samples. Data suggests transcription profiles in GUSTO maternal blood is similar to that from GTEx whole blood.

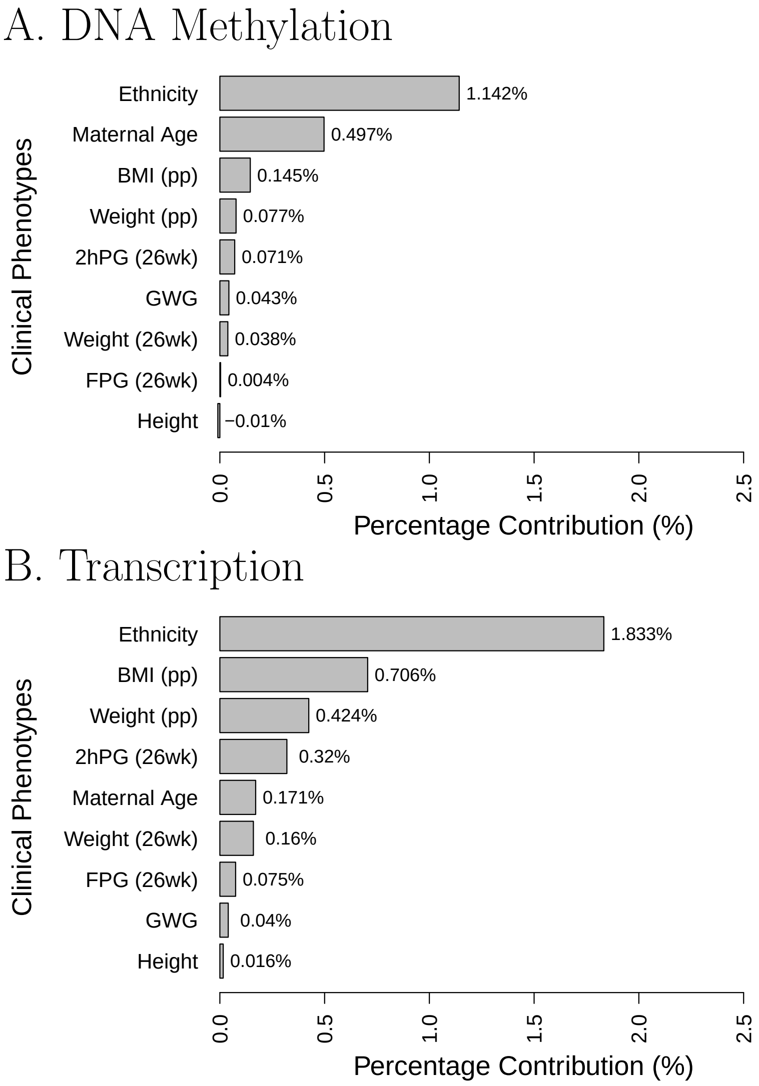

Supplementary Figure 4: Phenotypic Contribution to Variation. Eight clinical phenotypic measures were conducted to investigate the quantification of contribution of inter-individual variation cross platforms for DNA methylation and transcription. These eight measures were sample ethnicity, height, age, and measures at pre-pregnancy for body mass index (BMI (pp)), weight (Weight (pp)) , and measures at mid-term pregnancy, such as fasting and 2h post-75g-glucose challenge (FPG(26wk), 2hPG (26wk)), weight (weight (26wk)), gestational weight gain (GWG). **(A)** DNA methylation. **(B)** Transcription.

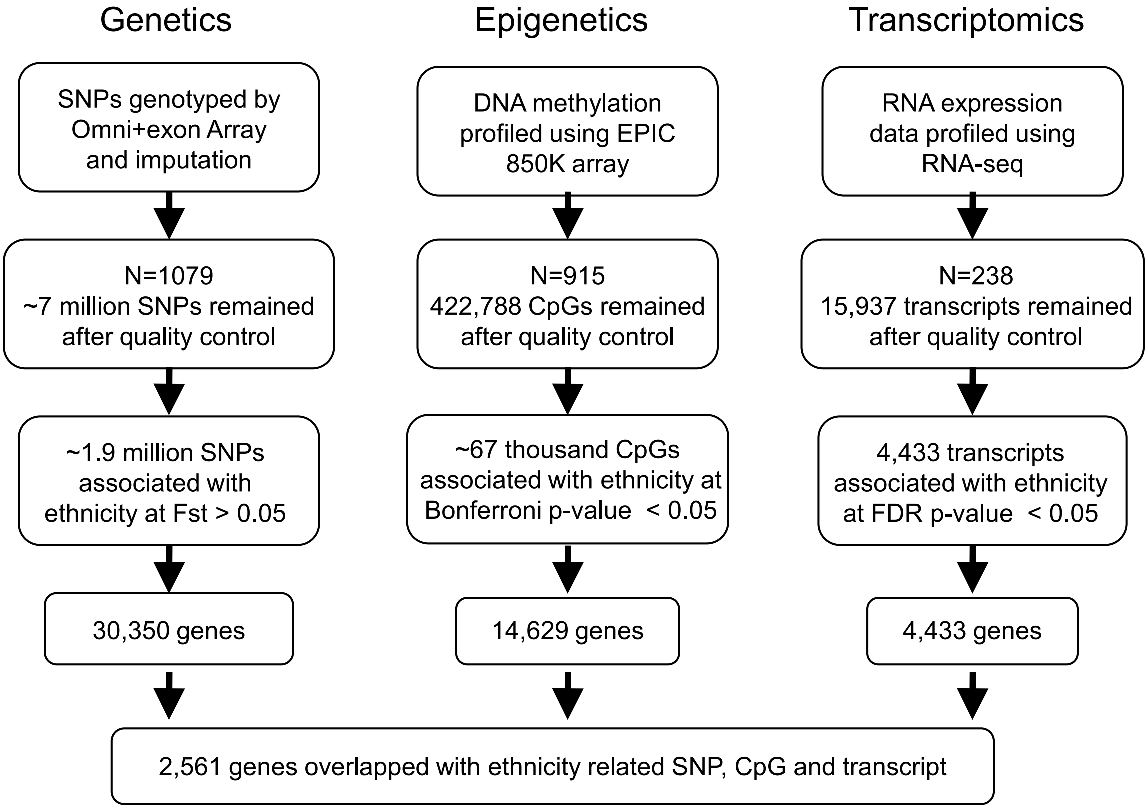

Supplementary Figure 5: The processing flow for genome-wide screen for ethnicity differentiations in genetics (left panel), epigenetics (middle panel) and transcriptomics (right panel).

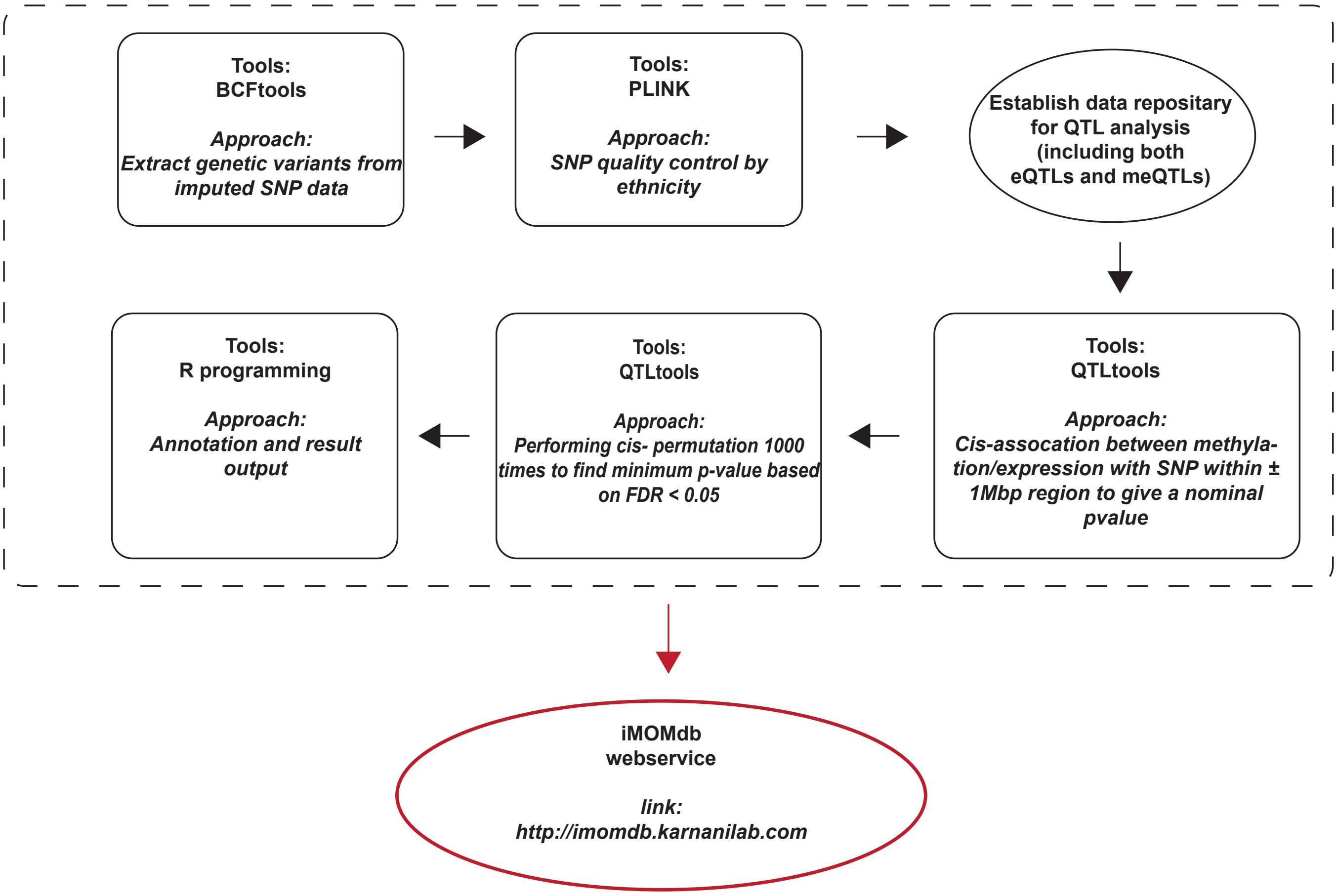

Supplementary Figure 6: The QTL mapping process flow. Data preprocessing and quality control were conducted by BCFtools and PLINK. The nominal p-values and permutation p-values of SNP-biomelecule associations were conducted by QTLtools.

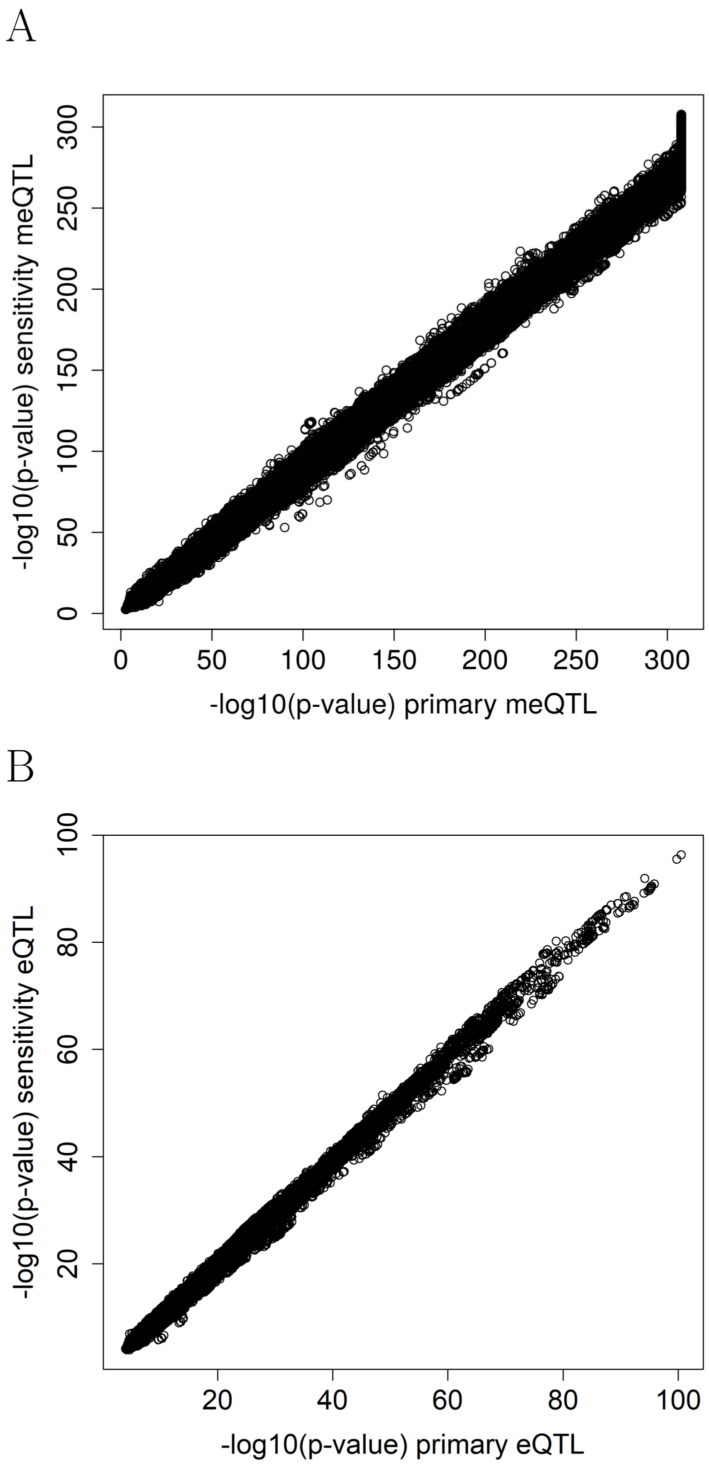

Supplementary Figure 7: The sensitivity studies of **(A)** meQTL and **(B)** eQTL. The x-axis is the the negative log10 transformed p-value of primary analysis and y-axis is the negative log10 transformed p-value of sensitivity analysis.

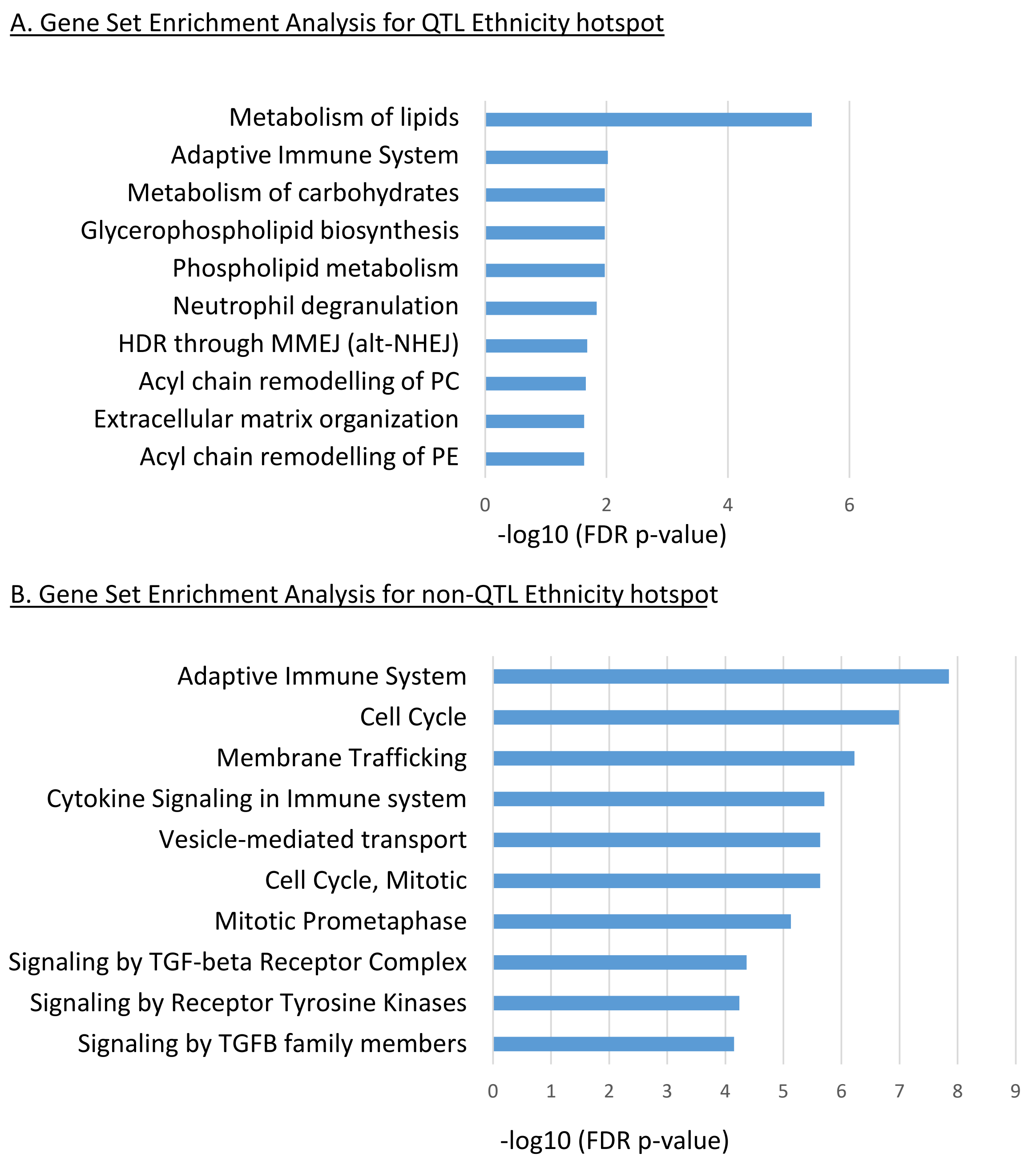

Supplementary Figure 8: Gene set enrichment analysis (GSEA). **(A)** The top 10 most significant gene sets from GSEA analysis on 395 ethnicity QTL hotspot genes. **(B)** The top 10 most significant gene sets from GSEA analysis on 520 non QTL ethnicity hotspot genes. The x-axis is the list of gene sets with less than 1000 genes. Y-axis is negative log10 transformed FDR p-value.
